# Single‐Session Combined Endodontic‐Periodontal Management of External Cervical Resorption: A 28‐Month Clinical and CBCT Follow‐Up

**DOI:** 10.1155/crid/9240567

**Published:** 2026-08-28

**Authors:** Mercedes Elvira Marval Figueroa, Maria Antonieta Mendez Rodulfo, Jose Francisco Gomez-Sosa

**Affiliations:** ^1^ Department of Periodontics, Universidad Central de Venezuela Facultad de Odontologia, Caracas, Venezuela; ^2^ Department of Endodontics, Nova Southeastern University College of Dental Medicine, Fort Lauderdale, Florida, USA

**Keywords:** bone regeneration, cone-beam computed tomography, guided tissue regeneration, incisor, root resorption

## Abstract

**Objectives:**

External cervical resorption (ECR) is a progressive condition that may compromise both periodontal and pulpal tissues. This report describes the outcome of a single‐session combined endodontic‐periodontal approach for the management of ECR in a previously treated maxillary central incisor.

**Case Presentation:**

A 41‐year‐old male presented with ECR affecting the maxillary left central incisor, associated with previous trauma, a sinus tract, and defective root canal treatment. Cone‐beam computed tomography (CBCT) was used for diagnosis, classification, and treatment planning. A single‐session approach combining endodontic retreatment and periodontal surgery was performed.

**Results:**

At 28 months, the tooth remained asymptomatic and functional, with clinical periodontal stability and radiographic periapical healing. However, no new bone formation was observed in the cervical buccal bone plate, and a residual probing depth of 5 mm persisted without bleeding or mobility.

**Conclusions:**

A single‐session combined approach may be a reasonable option for moderate ECR when adequate access and defect sealing are achievable. In this case, the outcome reflected disease control and structural stability rather than complete tissue regeneration.

## 1. Introduction

Root resorptions are characterized by loss of dental hard tissues due to odontoclastic activity. In primary teeth, this process is physiological and allows exfoliation and eruption of permanent successors. In the permanent dentition, resorption may occur internally within the canal system or externally from the root surface, and advanced lesions may extend coronally toward the crown. Root resorption in permanent teeth is typically pathological and irreversible [1].

External cervical resorption (ECR) has been associated with trauma, orthodontic treatment, and parafunctional habits, although its exact pathogenesis remains incompletely understood. Inflammation is considered a prerequisite for initiation, and the lesion progresses through invasion of cervical dentin by clastic cells and replacement by fibrovascular tissue [2–5].

Clinically, ECR may present as a pink discoloration, a cavitary defect near the cementoenamel junction, or a hard‐based lesion that may bleed on probing. Radiographically, it may appear as an irregular, asymmetric radiolucency with indistinct canal borders, sometimes with mixed radiopaque areas reflecting reparative activity [6].

The objective of treatment is to arrest the resorptive process by removing the affected tissue, eliminating clastic cell support, and sealing the defect with a biocompatible restorative material. Adequate visualization and access are critical to treatment success [1].

Periapical radiographs remain useful for diagnosis, but their two‐dimensional nature limits assessment of lesion extent. CBCT provides three‐dimensional visualization of lesion height, circumferential spread, and proximity to the root canal, and therefore is valuable in treatment planning and follow‐up. Heithersay and Patel classifications are useful frameworks, but both were originally developed for untreated teeth, so their application in previously treated teeth should be interpreted cautiously [6, 7].

Although several therapeutic strategies have been proposed for ECR, the literature still contains limited long‐term documentation of single‐session combined endodontic‐periodontal management with CBCT follow‐up. Therefore, this report is aimed at describing the diagnostic reasoning, treatment sequence, and longitudinal outcome of a moderate ECR case managed in a multidisciplinary single‐session approach.

## 2. Case Report

**Figure 1:**
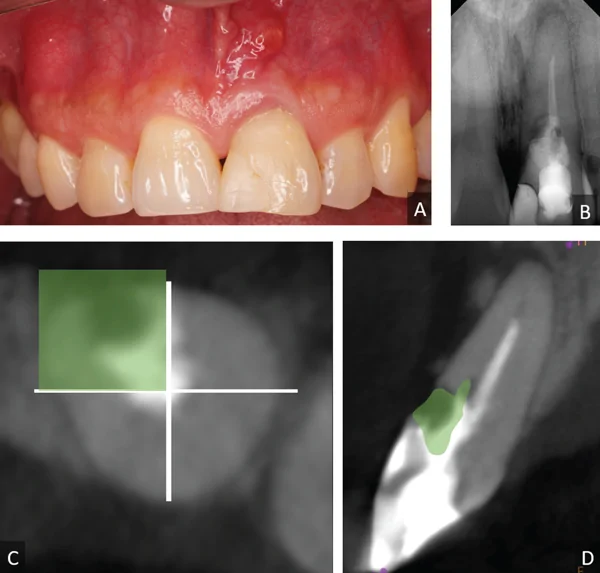
(A) Initial clinical presentation of the case. (B) Initial periapical radiographic image. (C,D) Axial and sagittal CBCT sections used to establish the classification according to the Patel [6] and Heithersay [4] criteria.

A 41‐year‐old male was referred for evaluation of the maxillary left central incisor. The patient reported a history of previous trauma to the tooth approximately 23 years before presentation and had undergone prior root canal treatment. The relevant medical history was noncontributory. Clinical examination revealed a localized sinus tract above the mucogingival line (Figure 1A), tenderness, and a probing depth of 3 mm at the cervical buccal aspect, with the remainder of the periodontal chart showing no alteration. Tooth mobility was not found.

Periapical radiography (Figure 1B) revealed a cervical radiolucent defect with apical inflammatory changes. CBCT demonstrated an ECR defect extending into the cervical and middle thirds of the root, with communication toward the root canal system. Based on the CBCT findings, the lesion was classified as Heithersay Class 2 and Patel 2Ap. CBCT acquisition was performed using Trophy Trophyan Smart 3D equipment, with a voxel size of 0.075 mm and a field of view of 5 × 5. (Figure 1C,D).

## 3. Endodontic Phase

**Figure 2:**
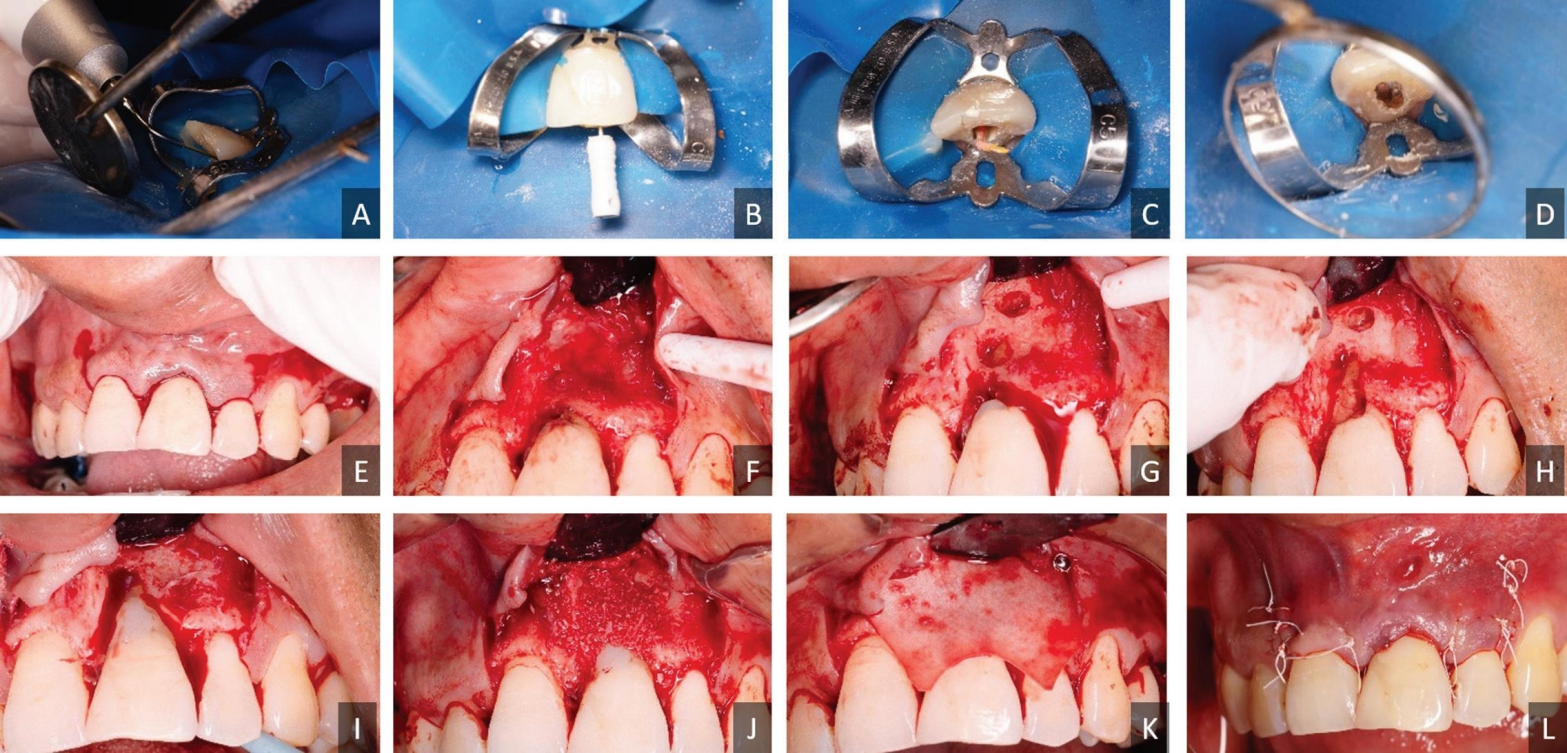
Endodontic retreatment and surgical management of the cervical resorptive defect. (A–D) Nonsurgical endodontic retreatment, including removal of the previous obturation material, canal preparation, irrigation, and re‐obturation. (E–H) Surgical exposure of the cervical resorptive defect after elevation of a full‐thickness mucoperiosteal flap, mechanical removal of granulation and resorptive tissue, refinement of the cavity walls, and osteotomy to improve access to the deeper portion of the lesion. (I) Restoration of the resorptive defect with composite resin under direct surgical visualization. (J–L) Placement of a porcine‐derived xenograft and resorbable membrane, followed by flap repositioning and suturing.

Because the tooth had a defective previous root canal treatment, nonsurgical endodontic retreatment was performed first under magnification (Opto DM 1591/03, Electrônica S/A, São Paolo, Brazil). After local anesthesia with 2% lidocaine and epinephrine and rubber dam isolation, coronal access was re‐established and the previous obturation material was removed (Figure 2A,B,C, and D). Canal cleaning and shaping were completed with E‐Flex Blue (Eighteeth, Changzhou Safari Medical Technology Co., Ltd, Jiangsu, China), and working length was determined with an electronic apex locator (Root ZX II, J Morita, Osaka, Japan) and confirmed radiographically. Apical preparation was completed to size 50/04.

Irrigation was performed with 5.25% sodium hypochlorite (Endo Solutions, Brasseler, Savannah, Georgia, United States) between instruments. Final irrigation included 5.25% sodium hypochlorite and 17% EDTA (MD‐Cleanser, Meta Biomed, Mülheim an der Ruhr, Germany), both ultrasonically activated according to a standardized sequence 1 min per each solution. The canal was dried with paper points and obturated using the continuous wave of condensation technique with gutta‐percha and AH Plus (Dentsply, Konstanz, Germany).

## 4. Surgical Phase

After completion of the endodontic phase, a full‐thickness mucoperiosteal flap was elevated with two vertical releasing incisions to expose the cervical resorptive defect. Granulation and resorptive tissue were carefully removed by mechanical curettage using hand instruments, followed by refinement of the cavity walls with a round diamond bur under copious irrigation, and osteotomy was performed to improve access to the deeper portion of the lesion. This approach allowed direct visualization of the defect and precise restoration under surgical conditions (Figure 2E,F,G, and H).

The resorptive defect was restored with composite resin (Micerium, Avegno, Italy) after etching with 37% phosphoric acid and application of a universal adhesive system (Zipbond, SDI, Victoria, Australia). Composite resin was selected because the defect was fully accessible, the lesion could be visualized directly, and precise contouring was required in the cervical esthetic zone. (Figure 2I).

A porcine‐derived xenograft (Corti − Oss granules ≤ 0.2–1 mm, Buenos Aires, Argentina) was placed within the defect and covered with a resorbable membrane (Corti‐Oss, Buenos Aires, Argentina). The flap was repositioned and sutured with nonresorbable sutures (Figure 2J,K, and L). The use of graft and membrane was intended as an adjunctive defect‐management strategy to support healing; however, the case design does not allow attribution of the observed radiographic changes solely to the grafting procedure.

Postoperative instructions were provided, and the patient received analgesic and antibiotic medication. Sutures were removed 15 days later. The patient was scheduled for serial follow‐up visits to assess symptoms, periodontal parameters, and radiographic changes.

## 5. Follow‐Up

**Figure 3:**
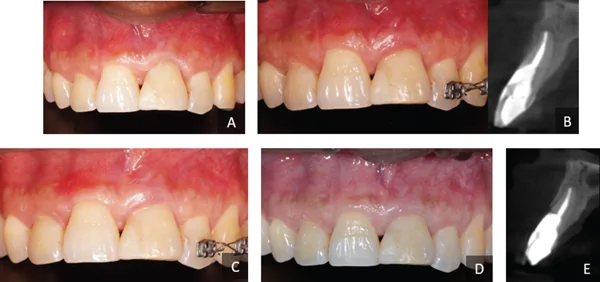
Re‐evaluation images showing periodontal health and sagittal section demonstrating apical bone regeneration at Tooth 21 and a hyperdense image in the cervical region consistent with restorative material. (A) 15‐day follow up. (B) 4‐month re‐evaluation. (C) 8‐month re‐evaluation. (D) 11‐month re‐evaluation. (E) 14‐month re‐evaluation.

At 6 weeks, (Figure 3A) the tooth was asymptomatic and showed no clinical signs of active inflammation. At 4 months, the periodontal tissues were clinically stable, with no bleeding on probing and no mobility; however, a localized buccal probing depth of 6 mm persisted. CBCT showed complete filling of the root canal system and periapical bone healing, but no new bone formation was observed in the cervical buccal bone plate (Figure 3B).

At 8 (Figure 3C) and 11 months (Figure 3D), the tooth remained asymptomatic, with healthy‐appearing periodontal tissues and a persistent localized probing depth of 5 mm at the mid‐buccal site, without bleeding or mobility. CBCT at 14 months (Figure 3E) confirmed resolution of the apical radiolucency and continued periapical repair.

**Figure 4:**
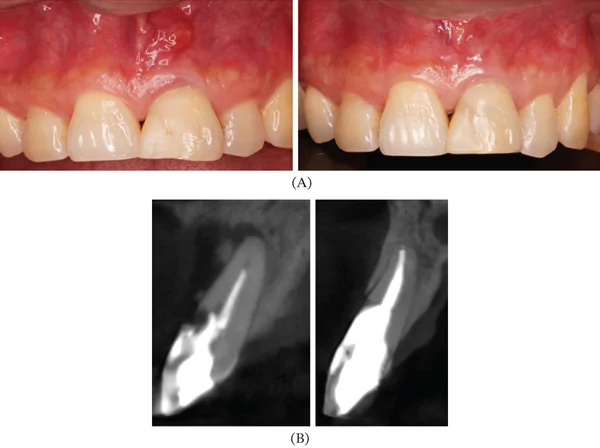
Comparison of the (A) initial and 28‐month follow‐up clinical and (B) CBCT images demonstrating lesion resolution and maintenance of periodontal and endodontic stability.

At 28 months, the tooth remained functional, asymptomatic, and stable. Clinical examination showed no bleeding on probing, no mobility, and no symptoms, but a localized residual probing depth of 5 mm persisted. CBCT demonstrated sustained periapical healing and structural stability of the restored defect, although no regeneration was evident in the cervical buccal bone plate. These findings were interpreted as disease control with residual periodontal sequela rather than complete regeneration (Figure 4).

## 6. Discussion

ECR remains diagnostically and therapeutically challenging because of its insidious onset, variable presentation, and complex etiologic associations. In this case, the lesion was linked to prior trauma and a defective root canal treatment, both of which may contribute to the development or persistence of resorptive pathology. CBCT was essential for diagnosis, classification, and treatment planning because conventional radiography alone would not have adequately defined lesion extent or communication with the canal system [7, 8].

The choice of a combined endodontic‐periodontal approach was based on the coexistence of a defective root canal treatment, apical pathology, and a surgically accessible cervical defect. The purpose of performing both phases in a single session was to reduce the risk of canal contamination through the resorptive defect, minimize treatment delay, and simplify patient management.

Mechanical debridement remains the most frequently described approach for Class 2 lesions and was the primary treatment strategy in the present case. Topical trichloroacetic acid has been widely reported as a useful adjunct in the management of invasive/ECR, where it promotes coagulation of the resorptive tissue and may facilitate its complete removal before restoration. However, in this case, the defect was surgically exposed, directly accessible under magnification, and communicated with the root canal system, allowing complete circumferential mechanical debridement under direct visualization until sound dentin was reached. Therefore, no topical chemical adjunct, including trichloroacetic acid or calcium hydroxide, was used. Sodium hypochlorite and EDTA were employed solely as standard intracanal irritants for endodontic disinfection and not as chemical agents for external resorptive tissue management. Nevertheless, the omission of topical trichloroacetic acid should be acknowledged as a limitation, as this adjunct remains a well‐supported option in the literature and may provide additional predictability in cases with more limited access or more complex lesion morphology [2, 9].

The restorative material in ECR must be selected according to access, defect geometry, and the ability to achieve a hermetic seal. Bioactive calcium silicate materials have advantages in many cervical defects, but composite resin can be a reasonable option when direct access is excellent and precise contouring is required in the cervical esthetic zone [2, 8, 10–12].

The use of xenograft and membrane should be interpreted cautiously. Although guided tissue regeneration was applied, this single‐case design does not permit causal attribution of the observed outcome to the grafting procedure. Accordingly, the clinical result should be described as stable healing with structural repair and persistent periodontal sequela, rather than proof of true regeneration.

The persistent localized probing depth deserves particular attention. Even in the absence of bleeding, mobility, or symptoms, residual pocketing indicates that periodontal healing was incomplete. For this reason, the final outcome should be framed as disease control rather than complete periodontal resolution.

The literature comparison should be interpreted with caution. Prior reports often describe staged management or shorter follow‐up intervals, whereas the present case provides longer CBCT‐based documentation after a single‐session combined approach. The practical learning point is therefore not that the strategy is revolutionary, but that it can achieve stable medium‐term control in selected moderate ECR cases when access, debridement, and sealing are adequate.

## 7. Clinical Implications

ECR requires three‐dimensional diagnosis and individualized treatment planning. In selected moderate lesions with combined pulpal and periodontal involvement, a single‐session multidisciplinary approach may be a reasonable option when direct access and adequate sealing are possible. Nevertheless, residual periodontal defects must be monitored closely because clinical stability does not necessarily mean complete tissue restoration.

## 8. Limitations

This report has several limitations inherent to its single‐case design. First, the absence of a topical chemical adjunct such as trichloroacetic acid means that the treatment cannot be directly compared with protocols that incorporate chemical devitalization of the resorptive tissue. Although complete mechanical debridement was achieved under direct visualization and no clinical or radiographic signs of recurrence were observed during the 28‐month follow‐up, the lack of adjunctive chemical treatment may affect the predictability of complete lesion inactivation in other cases. Second, the absence of quantitative CBCT volumetric analysis limits objective assessment of bone changes over time. Therefore, the conclusions should be restricted to the documented clinical behavior of this individual case and should not be generalized to all ECR presentations.

## 9. Conclusions

A combined endodontic‐periodontal approach performed in a single session may represent an effective and biologically sound strategy for the management of moderate ECR. Long‐term periapical healing and periodontal stability can be achieved. However, strict follow‐up remains essential, and treatment outcomes should be interpreted in terms of disease control and structural stability rather than complete tissue regeneration.

## Author Contributions

Mercedes Elvira Marval Figueroa: conceptualization, periodontal treatment, investigation, and writing—original draft. Maria Antonieta Mendez Rodulfo: supervision, data curation, clinical follow‐up, and writing—review and editing. Jose Francisco Gomez‐Sosa: endodontic treatment, supervision, methodology, and writing—review and editing.

## Funding

No funding was received for this manuscript.

## Ethics Statement

Written informed consent was obtained from the patient at the time of enrollment in a research study based on this clinical case. The patient authorized the use of clinical data and images for research and subsequent publication. All procedures were performed in accordance with ethical standards.

## Conflicts of Interest

The authors declare no conflicts of interest.

## Data Availability

The data supporting this case report are included within the article. Due to the nature of the study and patient confidentiality, no additional data are publicly available. Further details may be provided by the corresponding author upon reasonable request.

## References

[1] Patel S. , Saberi N. , Pimental T. , and Teng P. , Present Status and Future Directions: Root Resorption, International Endodontic Journal. (2022) 55, no. S4, 892–921, 10.1111/iej.13715, 35229320. 35229320 PMC9790676

[2] Bardini G. , Orrù C. , Ideo F. , Nagendrababu V. , Dummer P. , and Cotti E. , Clinical Management of External Cervical Resorption: A Systematic Review, Australian Endodontic Journal. (2023) 49, no. 3, 769–787, 10.1111/aej.12794, 37702252. 37702252

[3] Kikuta J. , Yamaguchi M. , Shimizu M. , Yoshino T. , and Kasai K. , Notch Signaling Induces Root Resorption via RANKL and IL-6 From hPDL Cells, Journal of Dental Research. (2015) 94, no. 1, 140–147, 10.1177/0022034514555364, 25376720. 25376720

[4] Heithersay G. S. , Clinical, Radiologic, and Histopathologic Features of Invasive Cervical Resorption, Quintessence International. (1999) 30, no. 1, 27–37, 10323156. 10323156

[5] Ferreira M. D. , Barros-Costa M. , Costa F. F. , and Freitas D. Q. , The Prevalence and Characteristics of External Cervical Resorption Based on Cone-Beam Computed Tomographic Imaging: A Cross-Sectional Study, Restorative Dentistry & Endodontics. (2022) 47, no. 4, 10.5395/rde.2022.47.e39, 36518614. PMC9715367 36518614

[6] Patel S. , Foschi F. , Mannocci F. , and Patel K. , External Cervical Resorption: A Three-Dimensional Classification, International Endodontic Journal. (2018) 51, no. 2, 206–214, 10.1111/iej.12824, 28746776. 28746776

[7] Heithersay G. S. , Invasive Cervical Resorption Following Trauma, Australian Endodontic Journal. (1999) 25, no. 2, 79–85, 10.1111/j.1747-4477.1999.tb00094.x. 11411085

[8] Fayad M. I. , Nair M. , Levin M. D. , Benavides E. , Rubinstein R. A. , Barghan S. , Hirschberg C. S. , and Ruprecht A. , AAE and AAOMR Joint Position Statement, Oral Pathology and Oral Radiology. (2015) 120, no. 4, 508–512, 10.1016/j.oooo.2015.07.033, 26346911. 26346911

[9] Heithersay G. S. , Treatment of Invasive Cervical Resorption: An Analysis of Results Using Topical Application of Trichloracetic Acid, Curettage, and Restoration, Quintessence International. (1999) 30, no. 2, 96–110, 10356561. 10356561

[10] Katarzyna L.-B. , Kinga K.-W. , Aleksandra K.-B. , and Monika S.-K. , Treatment of External Cervical Resorption and Its Late Complication: A Case Report, Iranian Endodontic Journal. (2022) 17, no. 1, 48–51, 10.22037/iej.v17i1.36672. 36703870 PMC9868990

[11] Ahmed N. , Mony B. , and Parthasarthy H. , External Cervical Resorption Case Report and a Brief Review of Literature, Journal of Natural Science, Biology and Medicine. (2014) 5, no. 1, 210–214, 10.4103/0976-9668.127336, 24678232. 24678232 PMC3961940

[12] Espona J. , Roig E. , Durán-Sindreu F. , Abella F. , Machado M. , and Roig M. , Invasive Cervical Resorption: Clinical Management in the Anterior Zone, Journal of Endodontics. (2018) 44, no. 11, 1749–1754, 10.1016/j.joen.2018.07.020, 30243659. 30243659

